# A Case of Myeloproliferative Neoplasm with BCR-FGFR1 Rearrangement: Favorable Outcome after Haploidentical Allogeneic Transplantation

**DOI:** 10.1155/2018/5724960

**Published:** 2018-12-06

**Authors:** Paola Villafuerte-Gutiérrez, Montserrat López Rubio, Pilar Herrera, Eva Arranz

**Affiliations:** ^1^Department of Hematology, Hospital Universitario Príncipe de Asturias, Alcalá de Henares, Madrid, Spain; ^2^Department of Hematology, Hospital Universitario Ramón y Cajal, Madrid, Spain; ^3^Cytogenetic Unit, Hospital Universitario de La Princesa, Madrid, Spain

## Abstract

Hematopoietic myeloproliferative neoplasms with FGFR1 rearrangement result in the 8p11 myeloproliferative syndrome that in the current Word Health Organization classification is designated as “myeloid and lymphoid neoplasm with FGFR1 abnormalities.” We report the case of a 66-year-old man who had clinical features that resembled chronic myeloid leukaemia (CML), but bone marrow cytogenetic and fluorescent in situ hybridization (FISH) studies showed t(8;22)(p11;q11) and BCR-FGFR1 fusion gene. He was initially managed with hydroxyurea, and given the aggressive nature of this disease, four months later, the patient underwent an allogeneic hematopoietic stem-cell transplantation (HSCT) from an HLA-haploidentical relative. Currently, HSCT may be the only therapeutic option for long-term survival at least until more efficacious tyrosine kinase inhibitors (TKIs) become available.

## 1. Introduction

Hematopoietic myeloproliferative neoplasms (MPN) with rearrangements of fibroblast growth factor receptor 1 (FGFR1) gene (located on chromosome 8p11) are uncommon and extremely aggressive entities. Translocations associated with this syndrome result in the fusion of the FGFR1 gene with various partners [1], resulting in ligand-independent FGFR activity. Patients with t(8;22)(p11;q11) and BCR-FGFR1 fusion gene have clinical features that resemble chronic myeloid leukaemia (CML) [2, 3].

It has been reported that MPN with a t(8;22) translocation and a chimeric BCR-FGFR1 fusion gene either present or rapidly transform into an acute leukaemia [3, 4], usually refractory to currently available chemotherapeutic regimens including tyrosine kinase inhibitors (TKIs) [2, 5]. No consensus on management is available for this condition. Allogeneic hematopoietic stem-cell transplantation (HSCT) is currently the only therapeutic option in BCR-FGFR1 MPN patients [6–8]. Patients who lack an HLA-matched sibling might benefit from alternative donors such as HLA-haploidentical relatives.

## 2. Case Presentation

We report the case of a 66-year-old man who was referred to us in January 2017 because of leukocytosis in a routine blood count. The patient was asymptomatic. On examination, there was no palpable lymphadenopathy, splenomegaly, or hepatomegaly.

Initial white blood cell (WBC) count was 36.7 × 10^9^/L, hemoglobin level 14.7 g/dl, and platelet count 600 × 10^9^/L. Peripheral blood (PB) examination showed leukocytosis with neutrophilia, metamyelocytes and myelocytes, basophilia, and no blasts. No eosinophilia and dysplasia were noted. LDH was 329 U/L (normal reference values 120–246 U/L).

**Figure 1:**
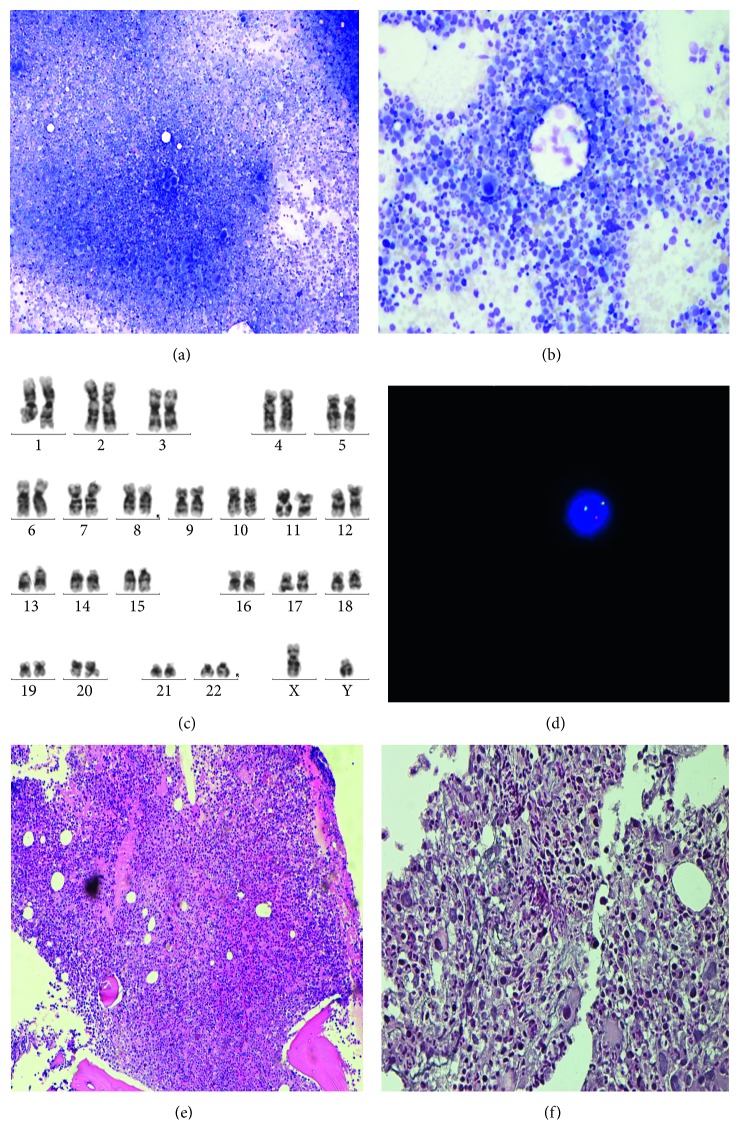
Bone marrow: (a, b) Low magnification of the bone marrow aspirate showing myeloid hyperplasia and hypolobated megakaryocytes; (c, d) cytogenetic analysis showed a 46,XY,t(8;22)(p11;q11)[19]/46,XY[1]. Fluorescence in situ hybridization showed the split of one of the two fusion signals indicating a chromosome breakage in the FGFR1 locus; (e) low magnification of the bone marrow biopsy showing hypercellular marrow (hematoxylin and eosin); (f) reticulin stain demonstrating a mild grade of fibrosis.

A bone marrow (BM) aspirate was hypercellular with myeloid hyperplasia; myeloid/erythroid ratio was increased to 9 : 1; blast count was 0.5% per nucleated marrow cells; and mild eosinophilia was evident. BM cytogenetics showed a 46,XY,t(8;22)(p11;q11)[19]/46,XY[1]. Fluorescence in situ hybridization (FISH) with LSI FGFR1 (8p11) Dual Color Break Apart (Abbott) showed the split of one of the two fusion signals indicating a chromosome breakage in the FGFR1 locus in 90% of 200 cells analyzed (Figure 1).

BCR-FGFR1 reverse-transcriptase polymerase chain reaction was not possible to carry out.

The patient was initially managed with hydroxyurea. He was offered an hematopoietic stem-cell transplantation considering the poor prognosis of patients with t(8;22)(p11.2;q11.2). A new BM aspirate was performed 6 weeks later due to anemia which confirmed the diagnosis. Cytogenetic analysis confirmed the presence of t(8;22) as the sole aberration in all 20 metaphases analyzed.

Four months later, the patient underwent an allogeneic HSCT from an HLA-haploidentical relative (haplo-HSCT). A nonmyeloablative conditioning regimen was used (cyclophosphamide, busulfan, and fludarabine). Five × 10^6^/kg CD34 cells were infused. Initial transplant course was unremarkable. Time to neutrophils >0.5 × 10^9^/L was 17 days, and time to platelets >20 × 10^9^/L was 20 days. No signs or symptoms of graft-versus-host disease (GVHD) were noted.

BM exams were performed regularly after HSCT. Cytogenetic remission as well as peripheral blood full-donor chimerism was documented from day 30. At the time of this writing (more than 8 months from HSCT), the patient remained clinically well without evidence of GVHD and in cytogenetic complete remission.

## 3. Discussion

Hematopoietic neoplasms with FGFR1 rearrangements are uncommon entities. Most recent WHO classification [16], has included them among “myeloid and lymphoid neoplasms associated with FGFR1 abnormalities (MLNAF),” which are characterized by reciprocal chromosome translocations involving FGFR1 gene located at chromosome 8p11 with a variety of fusion partner genes. One of the most frequently observed cytogenetic abnormalities is t(8;22) (p11.2;q11.2). The t(8;22) results in an in-frame fusion of FGFR1 on 8p11 and BCR on 22q11. The resultant fusion proteins activate tyrosine kinases which may result in the development of hematologic malignancies.

Patients with BCR-FGFR1 rearrangements may present with clinical and PB pictures which resemble chronic myeloid leukaemia [2, 4, 15, 17, 18]. The underlying BCR-FGFR1 rearrangement may thus be missed, so BM cytogenetic analysis remains the mainstay of diagnosis. In our case, cytogenetic analysis confirmed the presence of t(8;22) as the sole aberration, and no other additional chromosomal abnormalities were observed at diagnosis or during follow-up.

MLNAF may rapidly progress to CML-like blast crisis. Cases of B-acute lymphoblastic leukaemia (B-ALL), T lymphoblastic lymphoma, and acute myelogeneous leukaemia (AML) have also been reported [4, 9, 12–14, 19, 20].

Patients carrying the t(8;22)(p11;q11) and the subsequent BCR-FGFR1 fusion gene follow an aggressive course. There are a few reported patients who have been treated unsuccessfully with hydroxyurea [6], chemotherapy or TKIs [2, 5]. More potent TKIs, such as ponatinib which exhibits pan-FGFR inhibitory activity, may be of clinical benefit [10, 11]. Cardiovascular risk factors precluded the use of ponatinib in our case.

Myeloproliferative neoplasms with BCR-FGFRq rearrangement are usually refractory to chemotherapy, resulting in poor prognosis. We performed a literature search and found an additional 11 cases with BCR-FGFR1 rearrangement treated with allogeneic HSCT (Table 1), and to our knowledge, this case is the first report of a patient with a BCR-FGFR1 MPN treated with haploidentical HSCT. Haploidentical donors allow patients who lack a HLA-matched one to receive a HCST with outcomes which are much like those obtained with matched donors [21]. HSCT may be the only therapeutic option for long-term survival at least until more efficacious TKIs become available.

## Figures and Tables

**Table 1:** Characteristics of 12 patients with BCR-FGFR1 rearrangement treated with allo-HSTCT.

Case	Reference	Age (years)/sex	Diagnosis	Donor source	Response
1	Present report	65/M	MPN	Haploidentical HSCT	CCyR
2	Konishi et al., 2018 [8]	48/M	B-ALL/MPN	Mismatched unrelated, BM	CCyR
3	Montenegro et al., 2017 [3]	41/F	B-ALL/MPN	NR	Residual disease
4	Wang et al., 2016 [9]	56/F	B-ALL/MPN	Matched unrelated AHSCT	Remission for 5 months after AHSCT
5	Landberg et al., 2017 [10]	21/M	MPN	Mismatched unrelated AHSCT	Remission for 4 years after AHSCT
6	Khodadoust et al., 2015 [11]	47/M	Trilineage mixed-phenotype AL	Matched sibling allogeneic HSCT	Residual disease
7	Shimanuki et al., 2013 [12]	58/F	AL with dysplasia	Matched sibling allogeneic HSCT	Residual disease and subsequent relapse
8	Morishige et al., 2013 [7]	50/M	Trilineage AL/lymphoma	Cord blood	Remission for 2 years after AHSCT
9	Dolan et al., 2012 [6]	8/M	MDS/MPN	Unrelated AHSCT	Remission for 4.5 years after AHSCT
10	Haslam et al., 2012 [13]	21/M	B-ALL/MPN	Mismatched, unrelated AHSCT	Residual disease
11	Kim et al., 2011 [14]	59/M	Myeloid, T cell	AHSCT	CCyR
12	Patnaik et al., 2010 [15]	57/F	MPN	AHSCT	Remission for 42 months after AHSCT

Abbreviations: M: male; F: female; MPN: myeloproiferative neoplasm; B-ALL: B-cell acute lymphoblastic leukaemia; CCyR: complete cytogenetic response; AL: acute leukaemia; MDS: myelodysplastic syndrome; AHSCT: allogeneic hematopoietic stem-cell transplantation; BM: bone marrow; NR: Not reported.
